# Maternal and perinatal outcome of Ramadan fasting in women with gestational diabetes

**DOI:** 10.12669/pjms.39.2.7332

**Published:** 2023

**Authors:** Saba Abdullah, Shumaila, Saba Mughal, Mahwish Samuel, Nazli Hossain

**Affiliations:** 1 Saba Abdullah, MBBS., Postgraduate Resident, Dow University of Health Sciences, Karachi, Pakistan; 2 Shumaila, MBBS., Postgraduate Resident, Dow University of Health Sciences, Karachi, Pakistan; 3 Saba Mughal, M.Phil., Lecturer, School of Public Health, Dow University of Health Sciences, Karachi, Pakistan; 4 Mahwish Samuel, MBBS., Postgraduate Resident, Holy Family Hospital, Karachi, Pakistan; 5 Nazli Hossain, MBBS, FCPS, MBE., Professor, Dept. of Obstetrics & Gynecology Unit-I, Dow University of Health Sciences, Karachi, Pakistan

**Keywords:** Gestational Diabetes Mellitus, Pregnancy, Normoglycemia

## Abstract

**Objective:**

To compare maternal and perinatal outcome of Ramadan fasting during pregnancy in women with/without gestational diabetes.

**Methods:**

This prospective case-control study was conducted at Department of Obstetrics & Gynecology Unit 1 Ruth PKM Civil Hospital & Dow Medical College and Holy Family Hospital, Karachi during 1^st^ April to 31^st^ July, 2022. In this study normoglycemic pregnant women and those identified as gestational diabetes (n=52) on oral glucose tolerance test, who fasted during Ramadan were included. Women, on diet control or diet plus metformin were included in the study. Study questionnaire included demographic details, days of fasting, self-reported hypoglycemic episodes. Maternal outcomes included preterm birth, pregnancy induced hypertension. Perinatal outcome included hyperbilirubinemia, hypoglycemia, weight of placenta, and apgar score.

**Result:**

Eighty two women were included in the study, gestational diabetes (n=57) and normoglycemic (n=25). Average days of fasting were 16 ±9.0 days (range 5-30). Women with GDM were older (28.6 vs. 26.0 years, p-value=0.034), had raised levels of HbA1c (5.5 vs. 5.1, p-value=0.004), mean FBS (102.8 vs. 84.6 mg/dl, p-value <0.001), mean RBS (135.3 vs. 106.4 mg/dl, p-value <0.001) and had higher BMI at delivery (31.0 vs. 26.6 kg/m^2^, p-value=0.004). HbA1c (p-value=0.016) and head circumference of baby (p-value=0.038) were found lower in the group who fasted for more than 20 days among normoglycemic pregnant women. No other maternal and neonatal outcomes were found to be significantly affected by Ramadan fasting among pregnant women with/without GDM.

**Conclusion:**

Gestational diabetes do not affect maternal and perinatal outcome among pregnant women.

## INTRODUCTION

Ramadan fasting is an important pillar of Islam. Fasting during pregnancy is not obligatory and Muslim women are required to complete the count, at a later stage. Majority of the women prefer to complete their count during the holy month of Ramadan. Not only they find it easier but socially more convenient.^1^ Surveys have found that more than 50% of pregnant women prefer to fast during the month of Ramadan, despite religious exemption.^2^

There are around two billion Muslims in the world, with majority living in the Asia-Pacific region.^3^ Though there are guidelines and scientific opinions for Ramadan fasting and pregnancy,^4^ there is limited data available for maternal fasting and gestational diabetes. This is due to lack of literature and scientific data. In a small study, pregnant diabetic women, who fasted during Ramadan were compared with normoglycemic women. The investigators did not find any adverse effect on fetal development, or any significant effect on metabolic profile of diabetic women who fasted.^5^ In another study diabetic women on insulin, and gestational diabetes were compared for any adverse effect due to fasting. There was improvement in levels of glycosylated hemoglobin levels(HbA1C) and serum fructosamine in both groups of women, without any adverse fetal effects.^6^ The changes in glycosylated hemoglobin levels persisted after Ramadan. The authors suggested instead of complete ban on fasting among women on Insulin therapy, better collaboration with physician and blood glucose monitoring may be more helpful.

There is increased prevalence of gestational diabetes and diabetes mellitus among people of South-Asian origin. The prevalence of gestational diabetes for Pakistan is estimated to be around 14%.^7^ This translates into the fact that in considerable number of women Ramadan intersects pregnancy.

During the month of Ramadan, depending on the region, the duration of fasting is between 14-16 hours. This year, the month of Ramadan was observed in April-May, with 14-15 hours of continuous fasting. Both Suhoor, (pre sun-rise meal, when fast starts) and Iftar (post sun-set meal, when fast is broken) have been found to be associated with periods of hyperglycemia and hypoglycemia.

Fasting in women with gestational diabetes is not recommended, for adverse maternal and fetal outcome. But majority of women do not pay attention to the medical advice and continue to fast in the month of Ramadan. The aim of the study was to see the maternal and fetal outcome of Ramadan fasting in women with gestational diabetes.

## METHODS

This prospective study was conducted at the Dept. of Obstetrics & Gynecology Unit-I, Ruth Pfau KM Civil Hospital & Dow Medical College, and Holy Family Hospital, Karachi.

### Inclusion criteria:

- Singleton pregnancy,
- Diagnosis of gestational diabetes mellitus (either made for first time or already diagnosed on routine screening as GDM in current pregnancy),
- Pregnant gestational diabetic women on diet control or diet and metformin therapy,
- Pregnant women with normal glucose levels who fasted.

### Exclusion criteria:

- Multiple pregnancy,
- Insulin dependent diabetes mellitus,
- Anomalous baby and diagnosed intrauterine demise.

Pregnant women, diagnosed with gestational diabetes mellitus, registered with the antenatal clinic of the department or delivering with the unit were included in the study, after informed verbal consent. The study period was from April to July 2022. The diagnosis of gestational diabetes mellitus (GDM) is made on oral glucose tolerance test, carried out with 75gm glucose, between 24-28 weeks of gestation. Two abnormal glucose values indicated the presence of gestational diabetes mellitus. Patients diagnosed with GDM, on diet control only or those with diet control plus metformin were included in the study. Days of fasting were stratified as 1-10 days, 11-20 days and more than 20 days of fasting. This group was compared with women who were found normoglycemic on oral glucose tolerance test, and fasted during the month of Ramadan.

Demographic variables included maternal age, parity, family history of diabetes mellitus, body mass index at the start of pregnancy, weight gain during pregnancy. Also noted were history of continuous or intermittent fasting, and days of fasting. Maternal outcomes included pregnancy induced hypertension, preterm delivery, and mode of delivery. Hypertension was defined as new onset systolic blood pressure ≥140 mm hg, and diastolic blood pressure of ≥ 90 mm hg, with or without proteinuria. Preterm delivery was defined as delivery before 37 completed weeks of gestation. Perinatal outcome included fetal weight, apgar score at one and five minute, and weight of placenta at delivery. Around 3cc of blood was taken from neonate for serum blood sugar levels and serum bilirubin levels, within 24 hours of delivery. Hypoglycemia in newborn was defined as blood sugar levels ≤ 40mg/dl. Raised serum bilirubin levels was defined as baby requiring phototherapy or exchange transfusion. Also included were macrosomia (> 4.5kg) or small for gestational age (< 10^th^ percentile).

The details were collected on a predesigned questionnaire, and data was transferred on excel sheet for further analysis. Neonatal blood 3cc was collected for serum bilirubin and glucose levels within 24 hours of delivery. Weight of the placenta was done according to the standard technique, as elaborated in the previous study.^8^ Women were asked to maintain blood glucose levels, at least post Iftar, pre Iftar. Other biochemical markers included complete blood picture, glycosylated hemoglobin (HBA1c) levels before and after Ramadan. Women were also advised for self-monitoring of hypoglycemic events and to report in emergency in event of hypoglycemic events.

The study group was compared with fasting pregnant women, without the diagnosis of gestational diabetes mellitus. The study was approved by the Institutional Review Board of University. (IRB 2488/DUHS/Approval/2022/786).

### Sample size calculation and statistical analysis:

Sample size was calculated using OpenEpi calculator using 5% margin of error, 80% power, 95% confidence level, proportion of non-fasting group of pregnant women (13.9%) and odds ratio (6.6, as pregnant women with exposure of > 10 hours of Ramadan fasting more likely to develop hyperbilirubinemia in neonates).^9^ The total sample size came out to be 56 pregnant women with GDM.

Statistical Package for Social Sciences (SPSS) version 22.0 was used for data analysis. Descriptive statistics were reported as frequency (percentage) for categorical variables and mean (standard deviation) for numerical variables. Normality of continuous variables was checked by Shapiro-Wilk test. Independent t test/ Mann-Whitney U test/ Kruskal Wallis test were applied to check mean/ median differences of maternal and neonatal outcomes between the GDM and non-GDM groups and days of fasting. Chi-square test/ Fisher Exact test were used to check association between categorical outcome and independent variables. P-value <0.05 were considered to be statistically significant.

## RESULTS

A total of 82 pregnant women were included in this study who fasted in Ramadan during pregnancy. Average age of pregnant women was 27.8 ±5.4 years which ranged from 18 to 40 years whereas average BMI at start of pregnancy was 28.4 ±7.1 and at delivery was 29.7 ±6.7. Women fasted in Ramadan on average 16 ±9.0 days which ranged between five and 30 days. Among all, 57 (69.5%) women were diagnosed with GDM and 25 (30.5%) without GDM. Sixteen percent of the women experienced preterm delivery and sixty one percent underwent caesarean section. Neonatal blood glucose level was 67.2 ±14.1and serum bilirubin was 3.3 ±1.7 mg/dL whereas mean birth weight was 2.9 ±0.5 kg (Table-I).

**Table-I:** Descriptive statistics of maternal and neonatal characteristics (n=82).

Characteristics			Range
***Demographics- pregnant women***			
Maternal age (years)		27.8 ± 5.4	(18 - 40)
BMI at start (kg/m^2^)		28.4 ± 7.1	(18 - 45)
Days of fasting		15.8 ± 9.0	(5 - 30)
HbA1c (%)		5.3 ± 0.6	(4.0 - 7.3)
Mean FBS (mg/dL)		96.9 ± 14.7	(72 - 134)
Mean RBS (mg/dL)		125.8 ± 31.9	(78 - 232)
Consanguineous marriage, yes		33 (40.2)	
Family history of DM, yes		23 (28.0)	
***Maternal characteristics***			
Gestational age (weeks)		37.7 ± 1.4	(35 - 41)
BMI at delivery (kg/m^2^)		29.7 ± 6.7	(20 - 47)
GDM, yes		57 (69.5)	
***Treatment of GDM (n=57)***			
Diet alone		26 (45.6)	
Diet & Metformin		31 (54.4)	
***Mode of delivery***			
SVD/ Instruments		32 (39.0)	
LSCS		50 (61.0)	
Preterm delivery, yes		13 (15.9)	
***Neonatal characteristics***			
Weight of baby (kg)		2.9 ± 0.5	(1.9 - 4.0)
Length of baby (cm)		48.8 ± 3.1	(40 - 57)
Head circumference (cm)		33.4 ± 2.6	(29 - 53)
Mid upper arm circumference (cm)		9.8 ± 1.0	(8 - 13)
Weight of placenta (kg)		0.5 ± 0.1	(0.2 - 0.9)
Apgar score at 1-min		6.7 ± 0.8	(5 - 8)
Apgar score at 5-min		8.7 ± 0.5	(7 - 10)
Neonatal blood glucose level (mg/dL)		67.2 ± 14.1	(41 - 104)
Neonatal serum bilirubin (mg/dL)		3.3 ± 1.7	(1.3 - 9.9)
***Gender of baby***			
Male		39 (47.6)	
Female		43 (52.4)	

(Mean ± SD), (Min - Max) and n (%) are reported. BMI=Body Mass Index, FBS=Fasting blood sugar, RBS=Random blood sugar, GDM=Gestational diabetes mellitus, SVD=Spontaneous Vaginal Delivery, LSCS=Lower Segment Cesarean Section.

Comparisons of maternal and neonatal outcomes were made between groups of GDM and non-GDM. Those women who were diagnosed with GDM were older (28.6 vs. 26.0 years, p-value=0.034), had raised levels of HbA1c (5.5 vs. 5.1, p-value=0.004), mean FBS (102.8 vs. 84.6 mg/dl, p-value <0.001), mean RBS (135.3 vs. 106.4 mg/dl, p-value <0.001) and higher BMI at delivery (31.0 vs. 26.6 kg/m^2^, p-value=0.004) as compared to non-GDM pregnant women. Other maternal and neonatal outcomes did not show significant differences between the groups of GDM (Table-II).

**Table-II:** Maternal and neonatal outcomes by gestational diabetes mellitus (n=82)

Variables	GDM	Non-GDM	p-value*

	(n = 57)	(n = 25)	
***Demographics- pregnant women***			
Maternal age (years)	28.6 ± 4.9	26.0 ± 5.9	0.034
BMI at start (kg/m^2^)	29.1 ± 7.2	25.2 ± 6.1	0.099
Days of fasting	15.8 ± 8.8	15.7 ± 9.5	0.820
HbA1c (%)	5.5 ± 0.6	5.1 ± 0.5	0.004
Mean FBS (mg/dL)	102.8 ± 13.1	84.6 ± 9.5	< 0.001
Mean RBS (mg/dL)	135.3 ± 33.2	106.4 ± 17.3	< 0.001
***Family history of DM***			
Yes	19 (33.3)	4 (16.0)	0.108
No	38 (66.7)	21 (84.0)	
***Maternal outcomes***			
Gestational age (weeks)	37.6 ± 1.4	38.0 ± 1.3	0.178
BMI at delivery (kg/m^2^)	31.0 ± 6.8	26.6 ± 5.2	0.004
***Mode of delivery***			
SVD/ Instruments	19 (33.3)	13 (52.0)	0.111
LSCS	38 (66.7)	12 (48.0)	
***Preterm delivery***			
Yes	10 (17.5)	3 (12.0)	0.745
No	47 (82.5)	22 (88.0)	
***Neonatal outcomes***			
Weight of baby (kg)	2.9 ± 0.5	2.8 ± 0.4	0.635
Length of baby (cm)	48.8 ± 3.3	48.8 ± 2.5	0.714
Head circumference (cm)	33.1 ± 1.5	34.0 ± 4.1	0.385
Mid upper arm circumference (cm)	9.9 ± 1.0	9.6 ± 0.8	0.443
Weight of placenta (kg)	0.5 ± 0.1	0.5 ± 0.1	0.897
Apgar score at 1-min	6.8 ± 0.8	6.6 ± 0.7	0.547
Apgar score at 5-min	8.7 ± 0.5	8.6 ± 0.6	0.885
Neonatal blood glucose (mg/dL)	67.5 ± 13.8	66.3 ± 15.1	0.870
Neonatal serum bilirubin (mg/dL)	3.6 ± 1.9	2.7 ± 0.9	0.057
***Gender of baby***			
Male	26 (45.6)	13 (52.0)	0.594
Female	31 (54.4)	12 (48.0)	

(Mean ± SD) and n (%) are reported. BMI=Body Mass Index, FBS=Fasting blood sugar, RBS=Random blood sugar, GDM=Gestational diabetes mellitus, SVD=Spontaneous Vaginal Delivery, LSCS=Lower Segment Cesarean Section.

* p-value calculated by independent t test/ Mann Whitney test and by Chi-square test/ Fisher exact test.

Effect of Ramadan fasting on maternal and neonatal outcomes was examined separately among women with GDM and without GDM by further categorizing the groups into days of fasting (<11, 11-20, >20). It was noted that fasting was not significantly associated with any maternal and neonatal outcomes among pregnant women with GDM. However significant lower levels of HbA1c (p-value=0.016) and head circumference of baby (p-value=0.038) were found in the group who fasted for more than 20 days among non-GDM pregnant women. No other maternal and neonatal outcomes were found to be significantly affected by Ramadan fasting among pregnant women without GDM (Table-III).

**Table-III:** Maternal and neonatal outcomes stratified by gestational diabetes and days of fasting (n=82).

Variables	Women with GDM (n=57)			p-value*	Women without GDM (n=25)			p-value*
	
	< 11	11 - 20	> 20		< 11	11 - 20	> 20	
	
	(n = 24)	(n = 17)	(n = 16)		(n = 11)	(n = 6)	(n = 8)	
***Demographics- pregnant women***								
Maternal age (years)	28.6 ± 5.4	29.2 ± 4.8	28.1 ± 4.6	0.872	24.5 ± 6.0	28.3 ± 6.6	26.4 ± 5.3	0.322
BMI at start (kg/m^2^)	30.3 ± 7.4	27.2 ± 7.2	29.4 ± 7.2	0.475	28.2 ± 7.8	25.2 ± 3.8	23.3 ± 6.6	0.372
HbA1c (%)	5.7 ± 0.6	5.5 ± 0.7	5.3 ± 0.4	0.120	5.2 ± 0.5	5.4 ± 0.2	4.6 ± 0.4	0.016
Mean FBS (mg/dL)	104.2 ± 13.5	101.7 ± 14.9	101.8 ± 10.8	0.655	82.5 ± 7.2	87.2 ± 14.1	85.5 ± 9.0	0.755
Mean RBS (mg/dL)	141.1 ± 34.4	117.0 ± 16.6	143.6 ± 37.7	0.082	110.1 ± 18.4	101.0 ± 13.7	105.2 ± 18.7	0.573
***Maternal outcomes***								
Gestational age (weeks)	37.3 ± 1.5	37.7 ± 1.4	38.0 ± 1.3	0.273	37.7 ± 1.3	38.2 ± 1.7	38.5 ± 1.2	0.501
BMI at delivery (kg/m^2^)	31.8 ± 7.0	30.4 ± 6.4	30.4 ± 7.4	0.763	27.0 ± 5.4	25.9 ± 4.7	26.6 ± 5.7	0.859
***Treatment of GDM (n=57)***								
Diet alone	10 (41.7)	9 (52.9)	7 (43.8)	0.763		-		
Diet & Metformin	14 (58.3)	8 (47.1)	9 (56.2)			-		
***Mode of delivery***								
SVD/ Instruments	7 (29.2)	6 (35.3)	6 (37.5)	0.843	5 (45.5)	3 (50.0)	5 (62.5)	0.876
LSCS	17 (70.8)	11 (64.7)	10 (62.5)		6 (54.5)	3 (50.0)	3 (37.5)	
***Preterm delivery***								
Yes	5 (20.8)	4 (23.5)	1 (6.2)	0.448	1 (9.1)	1 (16.7)	1 (12.5)	0.999
No	19 (79.2)	13 (76.5)	15 (93.8)		10 (90.9)	5 (83.3)	7 (87.5)	
***Neonatal outcomes***								
Weight of baby (kg)	2.9 ± 0.5	2.9 ± 0.6	2.8 ± 0.4	0.923	2.8 ± 0.3	2.8 ± 0.6	3.0 ± 0.3	0.338
Length of baby (cm)	47.7 ± 3.7	49.9 ± 3.1	49.2 ± 2.5	0.298	49.3 ± 1.9	47.0 ± 2.3	49.3 ± 2.8	0.090
Head circumference (cm)	32.6 ± 0.9	33.5 ± 2.1	33.4 ± 1.3	0.055	33.3 ± 0.6	37.2 ± 7.8	32.5 ± 1.4	0.038
MUAC (cm)	9.8 ± 1.0	9.9 ± 1.1	9.9 ± 1.0	0.762	9.8 ± 0.6	9.5 ± 1.3	9.4 ± 0.9	0.625
Weight of placenta (kg)	0.5 ± 0.1	0.6 ± 0.1	0.5 ± 0.1	0.546	0.6 ± 0.1	0.5 ± 0.1	0.5 ± 0.1	0.070
Apgar score at 1-min	6.8 ± 0.8	6.6 ± 0.9	6.8 ± 0.8	0.399	6.6 ± 0.9	6.7 ± 0.5	6.6 ± 0.7	0.965
Apgar score at 5-min	8.7 ± 0.5	8.7 ± 0.4	8.6 ± 0.5	0.650	8.6 ± 0.8	8.6 ± 0.5	8.7 ± 0.4	0.942
Neonatal blood glucose (mg/dL)	65.0 ± 13.4	67.7 ± 12.7	71.0 ± 15.5	0.417	68.6 ± 18.1	67.0 ± 18.4	62.6 ± 6.7	0.984
Neonatal serum bilirubin (mg/dL)	3.6 ± 2.1	3.5 ± 1.6	3.6 ± 2.1	0.992	2.9 ± 1.1	2.6 ± 1.1	2.4 ± 0.7	0.642

(Mean ± SD) and n (%) are reported. BMI=Body Mass Index, FBS=Fasting blood sugar, RBS=Random blood sugar, GDM=Gestational diabetes mellitus, SVD=Spontaneous Vaginal Delivery, LSCS=Lower Segment Cesarean Section, MUAC=Mid upper arm circumference.

* p-value calculated by Kruskal Wallis test and by Chi-square test/ Fisher Exact test.

## DISCUSSION

This study aimed to find out maternal and perinatal outcome among normoglycemic and in women with gestational diabetes mellitus. Fasting itself is associated with episodes of metabolic disturbances. These are aggravated in women with diabetes. Both hypoglycemia and hyperglycemia are detrimental for maternal and perinatal health.

The average duration of fast varied between 15-16 hours. There is evidence of more hypoglycemia in women on diet±metformin, who fast during pregnancy. In our study, there was a significant difference between mean fasting and random blood glucose level among normoglycemic and gestational diabetes women. Studies from other parts of the world have also shown a decrease in mean blood glucose levels and glycosylated hemoglobin levels. Azlin et al., studied pregnant diabetic women with Type-1 diabetes mellitus and observed a decrease in mean blood glucose levels post Ramadan.^10^ There were no self- reported episodes of hypoglycemia requiring medical attention. In a study from Malaysia, including both Insulin dependent diabetes and GDM, no episodes of hypoglycemia were reported, among the study population.^6^ Women with gestational diabetes had a higher body mass index, and positive family history of diabetes mellitus. Though we did not have facility for continuous glucose monitoring, it has been suggested that without such monitoring of blood glucose levels, serious episodes of hypoglycemia can be missed. It has also been observed that women tend not to report about the hypoglycemic events which they encounter during fasting.^11^ Afandi et al conducted a study on pregnant gestational diabetes women in their second and third trimesters, either on diet or diet and metformin.^12^ These women were monitored with continuous blood glucose monitoring or self-blood glucose monitoring. The investigators found more incidence of hypoglycemia, in women with continuous glucose monitoring. Though, no adverse event or hospitalization were reported in both groups for hypoglycemia. In another study of 25 pregnant women with gestational diabetes, either on diet alone, diet plus metformin or on insulin therapy, significant reduction in blood glucose levels and HbA1c was observed during Ramadan, when compared to Pre-Ramadan.^13^ We did not measure HbA1c levels post Ramadan in our study group. But there was significant difference in the levels of HbA1c among gestational diabetic and normoglycemic women. Also there was increased prevalence of family history of diabetes mellitus in women with gestational diabetes.

Though the studies on pregnancy with gestational diabetes are very few, there are no studies on diabetic ketoacidosis during Ramadan fasting among pregnant women. In a systemic review and meta-analysis of more than 18,000 women exposed to Ramadan fasting, there was no evidence of low birth weight or preterm deliveries.^14^ Preterm delivery was also not seen among our study participants.

Fetal effects of maternal fasting can be anticipated, as fetus receives nutrition from mother directly through facilitated diffusion. In a retrospective cohort study from Turkyie, pregnant women who were found hypoglycemic (blood glucose level < 70mg/dl) on oral glucose tolerance test with 75-gm glucose, gave birth to lower birth weight babies with decreased length and head circumference.^15^ We also found decreased head circumference in our study participants, who were normoglycemic and fasted. Apart from this we did not find any evidence of hypoglycemia or raised bilirubin levels in our study group. Maternal fasting may induce a condition of stress in-utero for fetus, and has been found to be associated with adult hood diseases like hypertension.^16^ Large scale population-based studies are needed to study the long-term effects, of decreased head circumference, in later part of life. Neonatal apgar scores at one and five minutes were better in gestational diabetes women, but did not reach statistical significance.

### Limitations:

We did not take into account the caloric intake of carbohydrate and fat rich diet in both groups of women. Since there was self-monitoring of blood glucose levels, hypoglycemia may have been missed in the study group.

## CONCLUSION

Fasting is an important pillar of Islam. Gestational diabetes affects metabolic profile of pregnant women. Fasting in this group of women may be associated with maternal and fetal risks. Though we did not find any adverse maternal and fetal effects of maternal fasting among women with gestational diabetes, larger scale studies with blinded continuous glucose monitoring is much needed to provide robust guidelines to pregnant diabetic women and physicians.

### Authors Contribution:

**SA, S and MS:** helped in data collection and synopsis writing

**SM:** Did data analysis and contributed in write up

**NH:** Conceived idea, IRB approval and manuscript writing and is responsible for integrity of the study.
